# The relationship between the psychological stress of adolescents in school and the prevalence of chronic low back pain: a cross-sectional study in China

**DOI:** 10.1186/s13034-019-0283-2

**Published:** 2019-06-17

**Authors:** Qixiang Mei, Chunlin Li, Yue Yin, Qi Wang, Qiugen Wang, Guoying Deng

**Affiliations:** 1 Shanghai General Hospital, Shanghai Jiao Tong University School of Medicine, Shanghai, 200080 China; 2 Trauma Center, Shanghai General Hospital, Shanghai Jiao Tong University School of Medicine, No. 650 Xin Songjiang Road, Shanghai, 201620 People’s Republic of China

**Keywords:** Chinese college students, SCL-90, CLBP, Unhealthy mental states

## Abstract

**Background:**

Accumulating evidence supports an association between an unhealthy mental state and low back pain (LBP). However, the degree of the association between mental health and chronic low back pain (CLBP) in the general population is poorly understood. The objective of this study was to analyze the incidence of CLBP in Chinese college students and to examine the association between students’ unhealthy mental states and the prevalence of CLBP.

**Methods:**

This is a cross-sectional study. A total of 10,000 questionnaires were distributed in the second semester of the 2017–2018 academic year by the School of Medicine, Shanghai JiaoTong University. Eligible participants were students aged ≥ 18 years from randomly selected Chinese colleges. Participants completed a questionnaire survey that included items from the Symptom Checklist-90 (SCL-90) and items on demographic factors, LBP prevalence, quality of life at their university, study-related stress and interpersonal relationships. The evaluation of students’ mental states in the survey was divided into two major parts: direct and indirect indicators. A multivariate logistic regression model was mainly used to explore the relationship between CLBP and the students’ mental health.

**Results:**

There was a high incidence of CLBP in the college students. Multiple logistic regression analysis indicated that the risk of CLBP increased with increasing scores on the SCL-90, and a clinically unhealthy mental state (scores greater than 3) was significantly associated with CLBP (adjusted odds ratios for depression, anxiety, coercion, paranoia, and interpersonal sensitivity were 7.209, 6.593, 3.959, 4.465, and 4.283, respectively; p < 0.001). Participants who had poor living habits or uncomfortable campus lives and those who experienced heavy academic pressure also showed a higher positive association with CLBP compared with the full sample.

**Conclusions:**

Unhealthy psychological conditions, which may be attributed to unsatisfying school lives, excessive learning pressure, and uncomfortable interpersonal relationships, represent a risk factor for CLBP in college students.

**Electronic supplementary material:**

The online version of this article (10.1186/s13034-019-0283-2) contains supplementary material, which is available to authorized users.

## Introduction

Low back pain (LBP) is a common musculoskeletal health problem. It is nonspecific and self-limiting, and a subset of patients develop chronic low back pain (CLBP), defined as symptoms persisting for longer than 3 months [1]. LBP imposes a great financial burden on healthcare systems in many countries and has become a leading cause of disability [2]. The number of individuals with LBP is likely to increase substantially over the coming decades [3]. Thus, special attention must be paid to LBP.

LBP occurs at all ages and has a significant impact on the quality of life of children and adolescents [4, 5]. In addition, psychological strain due to heavy social pressure is challenging for young people. Studies have shown that college students are presently suffering from psychological pressure related to future employment, their family, and their studies, and they have difficulty addressing interpersonal relationships. At this stage, students often encounter psychological problems such as depression, anxiety, and interpersonal sensitivity [6, 7]. However, a poor mental state is also a contributing factor to the development of and recovery from LBP [8, 9]. Kamper et al. noted that multidisciplinary biopsychosocial rehabilitation interventions are more effective than conventional care and physical therapy for reducing pain and disability in patients with CLBP, which also confirms the important relationship between psychological factors and LBP [10].

Although the psychological problems of adolescents and the factors that influence them have gradually become research focus, research into the influence of psychological factors on the prevalence of CLBP is still relatively insufficient. Previous related research has focused on high school students in China [11]. However, Chinese college students also represent a large group of adolescents who need considerable attention. Therefore, it is necessary to investigate the psychological status of college students and to analyze the impact of psychological factors on the prevalence of CLBP.

This study will investigate the psychological experiences of college students in China through extensive sample surveys and will analyze the relationship between students’ responses and the prevalence of CLBP to provide a reference for improving the health of college students in the future.

## Materials and methods

### Study design

This cross-sectional study was designed to investigate the relationship between the psychological stress of adolescents in school and the prevalence of CLBP. Students aged 18 to 24 years were selected from colleges registered in China from January 2018 to June 2018 according to a cluster randomization method. A total of 50 colleges were randomly selected, and 200 students were randomly selected from each school.

### Ethical approval

All of the participants involved in the study provided their electronic written informed consent before being surveyed. This study followed the Helsinki Declaration. The study protocol was reviewed and approved by the Ethics Committee of Shanghai General Hospital, Shanghai Jiao Tong University, School of Medicine (Approval No. 2013KY002). Ethical approval for the study protocol was provided by the ethical boards of the School of Medicine, Shanghai Jiaotong University.

### Exclusion criteria

People with recent neck, shoulder, and lumbar injuries or a history of pain with obvious triggers were excluded. To ensure the validity of the data, questionnaires with incomplete answers, errors that were clearly not related to the questions or response options, and clear errors in logic were excluded before the analysis.

### Design of the questionnaire

Based on related literature and prior interviews, the questionnaire was designed and modified to capture the actual experiences of college students [12]. The questionnaire typically took less than 20 min to complete.

The questionnaire was divided into 3 parts. The first part included items on demographic factors, such as the respondents’ gender, grade, and professional information. The second part of the questionnaire investigated the prevalence of CLBP in college students. A diagnosis of LBP was made by obtaining participant information regarding the presence and frequency of back discomfort. We defined “chronic pain” as “pain lasting for over 6 h at a time or for short but frequent periods over 2–3 days more than 3 times in 3 months”. Data from participants who provided ambiguous answers were excluded. Students with severe symptoms were advised to visit a nearby hospital.

The third part of the questionnaire included items pertaining to the students’ mental status. The content was divided into direct indicators and indirect indicators. For the direct indicators, the 5 most representative dimensions of the SCL-90 were selected to assess the students’ mental state of college students; these dimensions were depression, anxiety, obsessive-compulsiveness, paranoid ideation and interpersonal sensitivity. The scores for each status and the related clinical condition were as follows: 1–1.99 indicated no evidence, 2–2.99 was considered slight evidence, 3–3.99 indicated subclinical symptoms, and 4–5 indicated clinical symptoms [12]. We considered the life status, learning pressure and interpersonal relationships of the college students to be indirect indicators of their mental states.

### Validation and reliability

A presurvey was conducted before the launch of the formal survey to ensure the validity and the logic of the questionnaire. Four hundred college students agreed to the interview. With reference to the presurvey, the survey questionnaire was modified to eliminate duplication and to remove factors that had little correlation with disease prevalence (measurement of sampling adequacy (MSA) < 0.50).

The study used standard questionnaires with a defined set of questions (a copy of the questionnaire is provided in Additional files 1, 2), and the logic of each question was evaluated in the presurvey to ensure that the survey participants could understand the questions and respond appropriately. The questionnaire was standardized using test–retest reliability and principal component analysis validity tests. Two weeks after the completion of the large-scale questionnaire survey, another 400 participants were randomly selected for a two-factor test–retest reliability study. The average test–retest reliability was 0.861, which was measured by the kappa statistic. The final Kaiser–Mayer–Olkin (KMO) index of the questionnaire was 0.815.

Fifty undergraduates from the Department of Clinical Medicine at the School of Medicine, Shanghai Jiao Tong University, were chosen to distribute and retrieve the questionnaires. These undergraduates were majoring in clinical medicine; they had a solid professional foundation and were trained in advance before the survey was administered. Low back pain (LBP) was defined as pain, muscle tension or stiffness, located below the costal margin and above the gluteal fold, with or without leg pain (sciatica), and most of them are not specific [13]. The participants received a popular science lecture that indicated the specific scope of LBP using diagrams of the human body and explained in detail the characteristics of the pain and the differences among postexercise soreness, menstrual pain in women, and posttraumatic pain.

### Data statistics

Data analysis was performed with SPSS 21.0 software (SPSS, Inc., Chicago, IL). Questionnaires with incomplete answers, errors that were clearly not caused by the questions or response options, and clear errors in logic were excluded. Multiple logistic regression analysis was used to examine all the risk factors, and those with p-values < 0.2 were extracted. A backward stepwise regression procedure was performed, and the threshold for variant removal was set at 0.05. The results are presented using odds ratios (ORs) and 95% confidence intervals (CIs). Statistical significance was indicated by a two-tailed *p* value < 0.05.

## Results

In this survey, a total of 10,000 questionnaires were delivered. We successfully retrieved 9453 (94.53%) and ultimately obtained 8664 valid questionnaires (86.64%). We excluded the unqualified and invalid questionnaires (i.e., those for which more than 15% of the answers did not reflect the participants’ intended response.

**Fig. 1:**
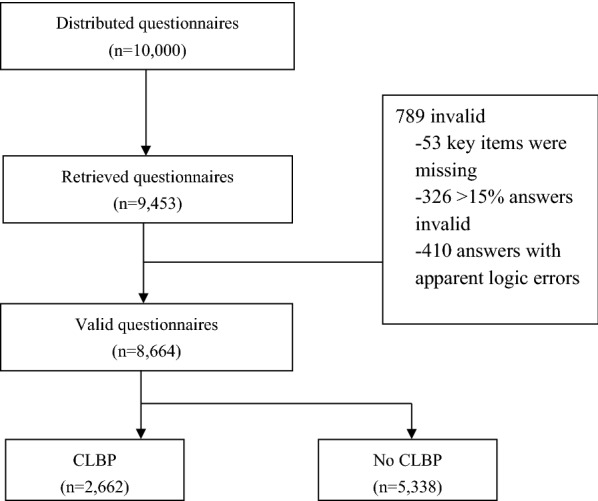
Distribution of questionnaires

Among the 789 unqualified questionnaires, 410 were excluded because they were far from complete, and 53 were excluded due to a lack of response to key questions (Fig. 1). The analysis of the remaining 326 incomplete questionnaires showed that the prevalence rates of CLBP were not significantly different from those indicated by the 8664 completed questionnaires (26.58% vs. 26.62%).

### Chronic low back pain and demographic information

The demographic characteristics of the participants are shown in Table 1. The respondents comprised 3949 males and 4715 females. The average age of the respondents was 19.95 ± 2.67 years. The overall incidence of CLBP in the college students was 26.62%. According to the survey results, the prevalence rates of CLBP were higher in female students than in male students (28.70% vs. 24.08%), and this difference was statistically significant.

**Table 1:** Prevalence of CLBP in college students by demographic factors

	N	Prevalence (%)	p-value	OR (95% CI)
Sex			< 0.001	
Male	3949	24.08	–	1
Female	4715	28.70	< 0.001	1.271 (1.154–1.400)
Major			< 0.001	
Engineering	3141	23.94	–	1
Arts	1320	26.67	0.054	1.155 (0.997–1.338)
Science	1371	24.36	0.761	1.023 (0.882–1.187)
Agriculture	76	39.47	0.002	2.072 (1.299–3.306)
Business	740	30.27	< 0.001	1.379 (1.155–1.646)
Medicine	1417	29.57	< 0.001	1.334 (1.159–1.535)
Other	599	32.55	< 0.001	1.533 (1.269–1.853)
Year of study			< 0.001	
Freshman, sophomore	6561	24.71	–	1
Junior, senior	1386	30.38	< 0.001	1.330 (1.170–1.510)
Master’s level	423	34.28	< 0.001	1.590 (1.290–1.958)
Doctoral level	294	40.48	< 0.001	2.072 (1.631–2.633)

OR, OR after univariate logistic regression; CI, confidence interval

Regarding major, agricultural students had the highest prevalence of CLBP (39.47%), and engineering students had the lowest prevalence (23.94%).

In terms of year of study, doctoral students had the highest prevalence of CLBP (40.48%), while freshman and sophomore students had the lowest prevalence (24.71%). Prevalence was positively correlated with the respondents’ year of study.

### Direct indicators of mental health and chronic low back pain in college students

Five dimensions of the SCL-90 were used as a direct indicator of the college students’ mental health status.

The questionnaire responses indicated that 34.63% of the respondents had depressive symptoms; of these, 1.28% reached clinical status, with the highest prevalence rate. A total of 22.20% of the respondents had anxiety symptoms; of these, 1.10% had clinical levels of anxiety, for a prevalence of 65.26%.

Coercion, paranoia, and interpersonal sensitivity can also contribute to the severity of CLBP. These issues were prevalent among 45.98%, 27.57%, and 36.2% of the students, respectively, and the prevalence of CLBP among the students experiencing each condition was 49.67%, 57.14% and 54.14%, respectively. We found that LBP in the respondents was often accompanied by mental illness, and the differences were statistically significant in all cases (p < 0.01) (Table 2).

**Table 2:** Prevalence of CLBP according to mental health status

	N	Prevalence (%)	p-value	OR (95% CI)
Depression			< 0.001	
None	5664	19.76	–	1
Slight	2323	36.63	< 0.001	2.348 (2.110–2.613)
Subclinical	566	46.82	< 0.001	3.576 (2.994–4.271)
Clinical	111	63.96	< 0.001	7.209 (4.867–10.680)
Anxiety			< 0.001	
None	6741	22.18	–	1
Slight	1535	39.35	< 0.001	2.277 (2.024–2.560)
Subclinical	293	49.49	< 0.001	3.438 (2.715–4.353)
Clinical	95	65.26	< 0.001	6.593 (4.305–10.097)
Obsession–compulsion			< 0.001	
None	4680	19.96	–	1
Slight	3080	31.40	< 0.001	1.835 (1.635–2.038)
Subclinical	751	43.81	< 0.001	3.127 (2.662–3.673)
Clinical	153	49.67	< 0.001	3.959 (2.860–5.478)
Paranoid ideation			< 0.001	
None	6275	23.00	–	1
Slight	1852	32.67	< 0.001	1.625 (1.450–1.820)
Subclinical	439	46.01	< 0.001	2.854 (2.344–3.474)
Clinical	98	57.14	< 0.001	4.465 (2.980–6.690)
Interpersonal sensitivity			< 0.001	
None	5525	21.61	–	1
Slight	2358	31.93	< 0.001	1.702 (1.528–1.895))
Subclinical	600	43.5	< 0.001	2.793 (2.348–3.322)
Clinical	181	54.14	< 0.001	4.283 (3.175–5.777)

CI, confidence interval; OR, odds ratio after univariate logistic regression

### Indirect indicators of mental health and chronic low back pain in college students

Campus life experiences, learning stress and interpersonal relationships strongly affect the mental status of college students and can be regarded as indirect indicators of their mental health (Tables 3, 4, 5).

**Table 3:** Prevalence of CLBP according to campus life experiences

	N	Prevalence (%)	p-value	OR (95% CI)
Alcohol use			< 0.001	
Seldom	7950	25.85	–	1
Occasionally	282	29.43	< 0.001	1.196 (0.922–1.553)
Sometimes	160	33.75	< 0.001	1.461 (1.049–2.036)
Often	174	30.46	< 0.001	1.256 (0.906–1.742)
Always	98	62.24	< 0.001	4.729 (3.134–7.137)
Smoking			0.001	
Seldom	5674	24.34	–	1
Occasionally	2196	27.96	< 0.001	1.207 (1.080–1.348)
Sometimes	637	36.89	< 0.001	1.817 (1.530–2.158)
Often	111	40.54	< 0.001	2.120 (1.444–3.111)
Always	46	67.39	< 0.001	6.424 (3.458–11.936)
Satisfaction with major			< 0.001	
Very satisfied	1403	22.38	–	1
Satisfied	3210	24.33	0.152	1.115 (0.961–1.295)
Neutral	2795	28.19	< 0.001	1.362 (1.172–1.582)
Dissatisfied	793	31.27	< 0.001	1.578 (1.298–1.919)
Very dissatisfied	463	37.80	< 0.001	2.107 (1.681–2.642)
Satisfaction with school			< 0.001	
Very satisfied	1491	21.40	–	1
Satisfied	3255	24.15	0.037	1.170 (1.009–1.356)
Neutral	2661	29.54	< 0.001	1.540 (1.327–1.788)
Dissatisfied	828	31.04	< 0.001	1.654 (1.364–2.004)
Very dissatisfied	429	36.83	< 0.001	2.142 (1.699–2.701)
Difficulty falling asleep (per week)			< 0.001	
< 1	4337	19.62	–	1
1–2	2758	31.18	< 0.001	1.856 (1.663–2.072)
> 3	726	36.09	0.165	2.313 (1.953–2.739)
Every day	843	39.50	< 0.001	2.675 (2.286–3.13)

CI, confidence interval; OR, odds ratio after univariate logistic regression

**Table 4:** Prevalence of CLBP according to study stress

	N	Prevalence (%)	p-value	OR (95% CI)
High expectations from parents	< 0.001			
Yes	5711	27.51	–	1
No	2953	24.89	0.009	0.873 (0.789–0.967)
Difficulty adapting to the pace of campus life	< 0.001			
Yes	2079	33.19	–	1
No	6585	24.84	< 0.001	0.655 (0.588–0.729)
Feeling that the people around them are better than they are	< 0.001			
Yes	5701	28.91	–	1
No	2963	22.21	< 0.001	0.702 (0.633–0.779)
Afraid of being unable to meet their goals	< 0.001			
Yes	6658	28.54	–	1
No	2006	20.24	< 0.001	0.635 (0.563–0.717)

OR, odds ratio after univariate logistic regression; CI, confidence interval

**Table 5:** Prevalence of CLBP according to interpersonal relationships

	N	Prevalence (%)	p-value	OR (95% CI)
Harmonious classmate relationships			< 0.001	
Yes	5674	23.81	–	1
Moderately	2908	31.46	< 0.001	1.469 (1.330–1.622)
No	82	48.78	< 0.001	3.047 (1.968–4.719)
Harmonious family relationships			< 0.001	
Yes	5041	22.87	–	1
Moderately	3287	30.61	< 0.001	1.487 (1.347–1.642)
No	336	43.75	< 0.001	2.623 (2.094–3.286)
Emotional life status			< 0.001	
Very good	1330	21.73	–	1
Good	2267	25.28	0.016	1.218 (1.037–1.431)
Moderate	3284	25.37	0.009	1.224 (1.051–1.425)
Bad	1140	31.93	< 0.001	1.690 (1.411–2.023)
Very bad	643	38.41	< 0.001	2.247 (1.829–2.759)

CI, confidence interval; OR, odds ratio after univariate logistic regression

Participants who had unsatisfying campus lives tended to suffer from CLBP (Table 3). The survey results showed that the prevalence of CLBP in patients with cigarette and alcohol addiction was as high as 62.24% and 67.39%, respectively. Students who were dissatisfied with their school and major had a higher prevalence rate of back pain. Additionally, the ORs of sleep quality and LBP increased linearly with increasing rates of insomnia. We can conclude that poor campus life experiences and poor personal habits are risk factors for CLBP (p < 0.001).

Learning stress may be associated with CLBP (Table 4). According to the collected data, 65.92% of the college students felt that their parents placed high expectations on them, and the prevalence of CLBP among these students was 27.51%. Moreover, 24.00% of the college students found it difficult to adapt to their current pace of life, and their CLBP prevalence rate reached 33.19%.

A total of 65.80% of the college students felt that most people around them were better than they were and that others were more diligent than they were, and 76.85% were afraid that they could not achieve their goals. The prevalence of CLBP in these two groups was 28.91% and 28.54%, respectively.

The data showed that the prevalence of LBP was higher in respondents with poor interpersonal relationships (Table 5). The respondents who were often in conflict with classmates and family members had a high prevalence of CLBP (48.78% and 43.75%, respectively). A total of 7.42% of the college students thought that their emotional life was a disaster, and their LBP prevalence rate was 38.41%, yielding an OR value of 2.247 (1.829–2.759). As the respondents’ emotional states continued to deteriorate, the prevalence of CLBP rose.

## Discussion

Our previous study showed that there is a strong correlation between the prevalence of chronic pain and academic pressure among adolescents in Shanghai (China) [11]. This study expands the research population to the general population of China and uses learning pressure as an indirect indicator of the psychological state of college students. This is the first study to employ cross-sectional analysis to explore the associations between the self-reported mental health of Chinese adolescents and the prevalence of CLBP. According to the survey results, the prevalence of CLBP in college students in China was 26.62%, which may be lower than that in the local area but is still high [11, 14].

Student mental health problems are a growing concern in colleges in many countries [15, 16]. Students experience many pressures when beginning school. Increased independence, exposure to new social situations, the maintenance of academic responsibilities and increased access to alcohol or drugs place tremendous pressure on students. The results of our study show that the mental problems faced by college students today may greatly affect the prevalence of CLBP.

The distribution of CLBP by sex is consistent with that of previous studies [3]. The prevalence of CLBP was higher in female students than in male students. This finding may have several explanations. First, CLBP is related to physiological changes in the menstrual cycle, and the difference between chronic pain and menstrual pain is difficult to identify [17]. Second, a lack of muscle strength can lead to improper sitting postures, which are more likely to cause CLBP [18]. Third, primary dysmenorrhea is common in women, and women are more susceptible than men to adverse emotions and are more likely to experience pain. Therefore, the symptoms of CLBP are more likely to occur in women [19].

People with more physically demanding workloads at their jobs are more likely to suffer from LBP [20]. Agricultural students may experience more outdoor learning and a is relatively heavier burden on their lower back; consequently, these students have a higher prevalence of CLBP than others. Multivariate analysis indicated a significant correlation between year of study and chronic pain: with increasing grade levels, the prevalence of CLBP increased. Senior students often face great pressure, especially in terms of entrance examinations and academic research, which causes them to remain at their desks for long periods of time. Research has shown that sitting time is an independent risk factor for LBP, and a longer sitting time may explain the increased prevalence of CLBP in this group [21]. In addition, pressure can increase students’ susceptibility to physical changes and make them more likely to complain of pain.

A recent systematic review noted that the Patient Health Questionnaire-15 (PHQ-15) and the Symptom Checklist-90 (SCL-90) are the most suitable questionnaires for large-scale studies [22]. These questionnaires are relatively short and have good psychometric attributes. Despite some controversy, the SCL-90 is widely used to evaluate the mental health of Chinese people and is the most commonly used scale for evaluating the subjective symptoms of college students for research and practice [23]. For this study, we selected the 5 most representative dimensions of the SCL-90 and used them to measure the mental state of college students. The results showed that depression, anxiety, coercion, paranoia, and interpersonal sensitivity were risk factors for the onset of CLBP, which is consistent with findings of a previous study [24].

Emotions such as depression and anxiety can lead to a decrease in patients’ pain thresholds; this reduces their tolerance of pain and increases their sensitivity [25], which subsequently increases the prevalence of self-assessed CLBP. In addition, more negative emotions often indicate fewer positive emotions. As negative mood states may be associated with the release of peripheral inflammatory cytokines that cause feelings of pain, positive mood states can activate the endogenous opioid system to release oxytocin to relieve the effects of pain [26, 27]. A more positive momentary mood is associated with reduced momentary pain and fewer restrictions [28]. In addition, CLBP can lead to psychological problems, such as depression and anxiety, which contribute to a vicious cycle and aggravate CLBP among many students [25, 29].

Unhealthy mental states, which may be due to poor campus life experiences, learning stress and adverse interpersonal relationships, may also be associated with CLBP.

An unsatisfying campus life tends to increase the risk of CLBP. Fujii et al. used the Somatic Symptom Scale-8 (SSS-8) and the EuroQol Five-Dimension (EQ-5D) questionnaire to analyze the association between the burden of physical symptoms and health-related quality of life in Japanese adults with CLBP (n = 3100), and they found that poor quality of life may aggravate somatic pain in patients with CLBP [30]. The results showed that among college students, those with bad personal habits, such as alcoholism and smoking, had a higher risk of illness than others, which is consistent with the findings of previous research [31]. Insomnia is not uncommon among college students [32]; decreased sleep quality affects academic performance [33], and significant correlations were found between sleep quality and obsessive–compulsive symptoms, somatization, depression, anxiety and overall symptoms [34]. We found that students with insomnia had a higher incidence of CLBP, which mirrored the findings of previous research [35]. However, LBP in turn promotes insomnia [36]. Poor emotional experiences are also associated with LBP, which indicates why dissatisfaction with one’s school and major may lead to greater sensitivity to pain [37].

The results of the study showed that excessive learning pressure is a risk factor for a high incidence of CLBP in college students. High expectations from parents, the fast pace of life, the feeling that others are working harder, the fear of not being able to achieve one’s goals, and the intense class competition create excessive pressure on college students. The Chinese education system emphasizes the importance of academic achievement. The mainstream ideology of the general public is to pursue good grades and obtain a good job. This ideology may burden Chinese adolescents with high levels of mental pressure.

Excessive learning pressure can lead to heavy schoolbags and sedentary, overweight students who engage in less exercise [38]. College students may develop chest kyphosis and tilting of the spine when they are sedentary, which may cause back injury [39]. Currently, college students’ schoolbags tend to be too heavy, which puts excessive pressure on the lower back [40]. In addition, excessive learning pressure indirectly reduces students’ exercise time, which in turn leads to back muscle fatigue [41]. All of these factors contribute to the occurrence of CLBP.

The results of the study show that interpersonal problems are also a risk factor for CLBP. Some college students have a bad relationship with classmates and parents or feel that their emotional life is unsatisfying. To some extent, all of these situations cause certain psychological and social problems, which affects the prevalence of LBP.

There are some limitations to our study. First, the SCL-90 mainly measures a person’s psychological state and neglects his or her ability to adjust during certain situations. In addition, people with strong psychological adjustment ability, will soon adjust their mental state to an appropriate level, even if they encounter discomfort. However, a person with poor psychological adjustment skills who is in a good position may present a better psychological state than a person with high psychological adjustment ability who is not in a good position. In practice, this means that someone with a high SCL-90 score may not be mentally disabled, while someone with a low SCL-90 score may be. Second, a cross-sectional study cannot establish causal relationships. Third, the reliability and validity were relatively low because there was no scale in the self-assessment questionnaire that measured levels of pain. LBP is nonspecific and is difficult to identify. Therefore, prior to the launch of the formal survey, we provided full illustrations and explanations of LBP to ensure the standardization of the questionnaire, and we found that differences in the severity of pain among different individuals were not significant and that cases of severe pain were rare. Fourth, students with chronic pain were more willing to complete the survey, and students with low moods were more likely to avoid the survey. In addition, the psychological state of depressed students was susceptible to implied influence. Fifth, although the data sample was large, results are always affected by bias and cannot always represent the true situation. Finally, the criteria, standards and methods applied to pain and chronic pain vary, and it is difficult to conduct studies that are more convincing than previous studies. Thus, the problem of bias exists to a certain extent.

To minimize the bias, we conducted a presurvey. Through communication with the participants, the questions were revised to be more objective and correct. A professional psychological consultant conferred with the survey participants to help them address their emotional problems.

Overall, there is a high incidence of mental problems and CLBP in Chinese college students, and the association between these factors is strong.

## Conclusion

Currently, Chinese college students face serious psychological problems, which are closely related to the high prevalence of CLBP. In addition, as indirect indicators of mental health, poor quality of campus life, excessive learning pressure and unsatisfying interpersonal relationships contribute to CLBP.

As a result, we recommend that further cohort studies explore the intrinsic link between self-reported psychological problems and CLBP, and we call for the dedication of more attention and care to the mental health of college students.

## Additional files

**Additional file 1.** Questionnaire (the English version).

**Additional file 2.** Questionnaire (the Original version).

## Data Availability

The datasets used and/or analyzed during the current study are not publicly available due to the need to protect individual privacy but are available from the corresponding author on reasonable request.
