# Supplementary material for: The relationship between the psychological stress of adolescents in school and the prevalence of chronic low back pain: a cross-sectional study in China
Source: Child Adolesc Psychiatry Ment Health. 2019 Jun 17;13:24. doi: 10.1186/s13034-019-0283-2 (PMC6580587; doi:10.1186/s13034-019-0283-2)
Supplement: Supplementary file 1 — Additional file 1. Questionnaire (the English version). [file 13034_2019_283_MOESM1_ESM.docx]

**Study on the Correlation between Psychological Status of College Students and their Neck，Shoulder and Back Pain**

**Part1 Basic Information**

What is your height (in cm) ____________ Weight is (in kg) _________

Your age is _________ [fill in the blank]*

What is your gender? [single choice] *

| ○Male | ○Female |
| --- | --- |

What is your permanent residence before entering the university? [single choice]*

| ○Beijing, Shanghai, guangzhou and shenzhen |
| --- |
| ○Provincial capital |
| ○Second-tier city |
| ○Third-tier city |
| ○Rural |

What is your university and year? [fill in the blank] *

_________________________________

What is your major? [single choice] *

| ○Engineer | ○Arts | ○Science | ○Agricultural |
| --- | --- | --- | --- |
| ○Business | ○Medicine | ○Others |  |

What is your accommodation status during your study? [single choice] *

| ○Dormitory |
| --- |
| ○Rents |
| ○Stay with family or friends |

**Part 2 Chronic pain and its extension** (except for the following discomfort symptoms after strenuous exercise)

Rarely: < once a month;

Occasionally: 1-3 times a month;

Sometimes: 1-3 times a week;

Often: > 3 times a week;

Always: every day

How often do you have neck and shoulder pain? *

| ○Rarely |
| --- |
| ○Occasionally |
| ○Sometimes |
| ○Often |
| ○Always |

How often do you have low back pain *

| ○Rarely |
| --- |
| ○Occasionally |
| ○Sometimes |
| ○Often |
| ○Always |

What is the intensity of your symptoms? [single choice] *

| ○Serious impact on normal life |
| --- |
| ○Slight impact on normal life |
| ○No impact on normal life |
| ○None of the above |

How often do you smoke? [single choice] *

| ○Rarely |
| --- |
| ○Occasionally |
| ○Sometimes |
| ○Often |
| ○Always |

How often do you drink alcohol? [single choice] *

| ○Rarely |
| --- |
| ○Occasionally |
| ○Sometimes |
| ○Often |
| ○Always |

How long do you usually stay at work and study in a day? (cumulative total time) [single choice] *

| ○<1h |
| --- |
| ○1h-3h |
| ○3h-5h |
| ○5h-10h |
| ○>10h |

**Part 3 Mental health**
 (respondents can evaluate their mental health status through this section, and the assessment results will be displayed after completion of the questionnaire.)

In your recently life (nearly three months), whether you feel the following condition and according to the situation and select the corresponding options to be described

None - there is no this kind of condition;

Mild -- with this condition but mild

Moderate -- with this condition and evident

Severe – with evident condition and disturb your normal life

Very severe-- severely disturb your normal life

Declining interest in sexual orientation [single choice]*

| ○None | ○Mild | ○Moderate | ○Severe | ○Very severe |
| --- | --- | --- | --- | --- |

Feeling lower energy [single choice]*

| ○None | ○Mild | ○Moderate | ○Severe | ○Very severe |
| --- | --- | --- | --- | --- |

Have the idea of taking your own life [single choice]*

| ○None | ○Mild | ○Moderate | ○Severe | ○Very severe |
| --- | --- | --- | --- | --- |

Easy to cry [single choice] *

| ○None | ○Mild | ○Moderate | ○Severe | ○Very severe |
| --- | --- | --- | --- | --- |

Feeling cheated, tricked, or someone trying to catch you [multiple choice] *

| ○None | ○Mild | ○Moderate | ○Severe | ○Very severe |
| --- | --- | --- | --- | --- |

Always blame yourself. *

| ○None | ○Mild | ○Moderate | ○Severe | ○Very severe |
| --- | --- | --- | --- | --- |

Feeling lonely [single choice] *

| ○None | ○Mild | ○Moderate | ○Severe | ○Very severe |
| --- | --- | --- | --- | --- |

Feeling depressed [single choice] *

| ○None | ○Mild | ○Moderate | ○Severe | ○Very severe |
| --- | --- | --- | --- | --- |

Excessive worry [single choice]*

| ○None | ○Mild | ○Moderate | ○Severe | ○Very severe |
| --- | --- | --- | --- | --- |

Not interested in everything [single choice] *

| ○None | ○Mild | ○Moderate | ○Severe | ○Very severe |
| --- | --- | --- | --- | --- |

Feel hopeless [single choice] *

| ○None | ○Mild | ○Moderate | ○Severe | ○Very severe |
| --- | --- | --- | --- | --- |

Finding everything difficult [single choice] *

| ○None | ○Mild | ○Moderate | ○Severe | ○Very severe |
| --- | --- | --- | --- | --- |

Feeling worthless [single choice]*

| ○None | ○Mild | ○Moderate | ○Severe | ○Very severe |
| --- | --- | --- | --- | --- |

Feeling nervous, uneasy [single choice]*

| ○None | ○Mild | ○Moderate | ○Severe | ○Very severe |
| --- | --- | --- | --- | --- |

Shivering for no reason [single choice] *

| ○None | ○Mild | ○Moderate | ○Severe | ○Very severe |
| --- | --- | --- | --- | --- |

Sudden unprovoked fear [single choice] *

| ○None | ○Mild | ○Moderate | ○Severe | ○Very severe |
| --- | --- | --- | --- | --- |

Heart beats too fast [single choice] *

| ○None | ○Mild | ○Moderate | ○Severe | ○Very severe |
| --- | --- | --- | --- | --- |

Repeated fear or horror[single choice] *

| ○None | ○Mild | ○Moderate | ○Severe | ○Very severe |
| --- | --- | --- | --- | --- |

Feeling on pins and needles[single choice]*

| ○None | ○Mild | ○Moderate | ○Severe | ○Very severe |
| --- | --- | --- | --- | --- |

Feeling afraid [single choice] *

| ○None | ○Mild | ○Moderate | ○Severe | ○Very severe |
| --- | --- | --- | --- | --- |

Something that feels familiar becomes unfamiliar or unreal [single choice] *

| ○None | ○Mild | ○Moderate | ○Severe | ○Very severe |
| --- | --- | --- | --- | --- |

Feeling the need to get things done quickly [single choice] *

| ○None | ○Mild | ○Moderate | ○Severe | ○Very severe |
| --- | --- | --- | --- | --- |

Unnecessary thoughts or words swirl around in your mind. [single choice]*

| ○None | ○Mild | ○Moderate | ○Severe | ○Very severe |
| --- | --- | --- | --- | --- |

Poor memory [single choice]*

| ○None | ○Mild | ○Moderate | ○Severe | ○Very severe |
| --- | --- | --- | --- | --- |

Worrying about being well groomed [single choice]*

| ○None | ○Mild | ○Moderate | ○Severe | ○Very severe |
| --- | --- | --- | --- | --- |

Finding it difficult to complete the task. [single choice] *

| ○None | ○Mild | ○Moderate | ○Severe | ○Very severe |
| --- | --- | --- | --- | --- |

Things must be done slowly to make sure they are done correctly. [single choice] *

| ○None | ○Mild | ○Moderate | ○Severe | ○Very severe |
| --- | --- | --- | --- | --- |

Things must be double checked. [single choice]*

| ○None | ○Mild | ○Moderate | ○Severe | ○Very severe |
| --- | --- | --- | --- | --- |

Hard to make a decision. [single choice]*

| ○None | ○Mild | ○Moderate | ○Severe | ○Very severe |
| --- | --- | --- | --- | --- |

Feeling empty mind. [single choice] *

| ○None | ○Mild | ○Moderate | ○Severe | ○Very severe |
| --- | --- | --- | --- | --- |

Can't concentrate. [single choice]*

| ○None | ○Mild | ○Moderate | ○Severe | ○Very severe |
| --- | --- | --- | --- | --- |

Must wash hands, count or touch something repeatedly. [single choice] *

| ○None | ○Mild | ○Moderate | ○Severe | ○Very severe |
| --- | --- | --- | --- | --- |

Blame someone else for the trouble. [single choice] *

| ○None | ○Mild | ○Moderate | ○Severe | ○Very severe |
| --- | --- | --- | --- | --- |

Most people can't be trusted. [single choice]*

| ○None | ○Mild | ○Moderate | ○Severe | ○Very severe |
| --- | --- | --- | --- | --- |

Feeling that someone is watching you and talking about you. [single choice]*

| ○None | ○Mild | ○Moderate | ○Severe | ○Very severe |
| --- | --- | --- | --- | --- |

Some thoughts or ideas that others don't have. [single choice] *

| ○None | ○Mild | ○Moderate | ○Severe | ○Very severe |
| --- | --- | --- | --- | --- |

Feeling that your performance had not been properly evaluated. [single choice] *

| ○None | ○Mild | ○Moderate | ○Severe | ○Very severe |
| --- | --- | --- | --- | --- |

Feeling like someone is trying to take advantage of you. [single choice] *

| ○None | ○Mild | ○Moderate | ○Severe | ○Very severe |
| --- | --- | --- | --- | --- |

Blaming others for perfection [multiple choice]*

| ○None | ○Mild | ○Moderate | ○Severe | ○Very severe |
| --- | --- | --- | --- | --- |

Feeling shy and uncomfortable with the opposite sex [single choice] *

| ○None | ○Mild | ○Moderate | ○Severe | ○Very severe |
| --- | --- | --- | --- | --- |

Feelings are easily hurt [single choice] *

| ○None | ○Mild | ○Moderate | ○Severe | ○Very severe |
| --- | --- | --- | --- | --- |

Feeling that others do not understand you, do not sympathize with you [single choice] *

| ○None | ○Mild | ○Moderate | ○Severe | ○Very severe |
| --- | --- | --- | --- | --- |

Feeling that people are unfriendly to you and don't like you[single choice]*

| ○None | ○Mild | ○Moderate | ○Severe | ○Very severe |
| --- | --- | --- | --- | --- |

Feeling inferior to others[single choice]*

| ○None | ○Mild | ○Moderate | ○Severe | ○Very severe |
| --- | --- | --- | --- | --- |

Feeling uncomfortable when people look at you or talk about you[single choice]*

| ○None | ○Mild | ○Moderate | ○Severe | ○Very severe |
| --- | --- | --- | --- | --- |

Feeling sensitive to others [single choice] *

| ○None | ○Mild | ○Moderate | ○Severe | ○Very severe |
| --- | --- | --- | --- | --- |

Uncomfortable to eat in public [single choice] *

| ○None | ○Mild | ○Moderate | ○Severe | ○Very severe |
| --- | --- | --- | --- | --- |

**Part 4 Other pressures**

Do you have a clear plan for the future? [single choice]*

| ○No plan | ○Some vague ideas | ○A general direction | ○A clean plan |
| --- | --- | --- | --- |

How satisfied are you with your current school? [single choice]*

| ○Very satisfied | ○Satisfied | ○General | ○Satisfied | ○Very unsatisfied |
| --- | --- | --- | --- | --- |

How satisfied are you with your current major? [single choice] *

| ○Very satisfied | ○Satisfied | ○General | ○Satisfied | ○Very unsatisfied |
| --- | --- | --- | --- | --- |

Do you get along well with your classmates? [single choice] *

| ○Very harmonious, almost no conflict | ○Generally, there are occasional conflicts | ○Poor, frequent conflict |
| --- | --- | --- |

Do you get along well with your family (parents, siblings)? [single choice] *

| ○Very harmonious, almost no conflict | ○Generally, there are occasional conflicts | ○Poor, frequent conflict |
| --- | --- | --- |

Do you think your parents have high expectations of you [single choice] *

| ○Yes | ○No |
| --- | --- |

What do you think of your living standard? [single choice] *

| ○Rich | ○Balance | ○Insufficient. | ○Short |
| --- | --- | --- | --- |

Are you struggling to adjust to your current pace of life? [single choice]*

| ○Yes | ○NO |
| --- | --- |

How many hobbies do you have? (painting, photography, film, reading, etc.) [single choice]*

| ○1-2 | ○3-5 | ○More than 5 |
| --- | --- | --- |

Do you think time is pressing and not enough [single choice]*

| ○Yes | ○No |
| --- | --- |

How often do you have difficulty falling asleep? [single choice] *

| ○Almost everyday | ○Four to five times a week | ○Once or twice a week | ○Almost not |
| --- | --- | --- | --- |

How many clubs or student organizations have you joined? [single choice] *

| ○1 | ○2 | ○More than 3 | ○None |
| --- | --- | --- | --- |

What do you think of your emotional life? [single choice]*

| ○Very good | ○Good | ○General | ○Poor | ○Very poor |
| --- | --- | --- | --- | --- |

Do you feel that most people around you are better and work harder than you? [single choice] *

| ○Yes | ○No |
| --- | --- |

Are you afraid of not reaching your goals? [single choice]*

| ○Yes | ○No |
| --- | --- |

Do you have any symptoms or signs of hair loss? [single choice]*

| ○Yes | ○No |
| --- | --- |

How do you feel about the competition among your classmates? [single choice] *

| ○Very fierce | ○Fierce | ○General | ○No feeling | ○No care |
| --- | --- | --- | --- | --- |

Where do you spend your spare time? [single choice] *

| ○Dormitory | ○Library, | ○Stadium | ○Outside the school |
| --- | --- | --- | --- |

Do you think your grades are satisfactory? (objectively speaking, don't be modest) [single choice] *

| ○Very satisfied | ○Satisfied | ○General | ○Satisfied | ○Very unsatisfied |
| --- | --- | --- | --- | --- |

Do you have enough time for recreational activities in your spare time? [single choice]*

| ○Yes | ○No |
| --- | --- |

How often do you exercise per week? [single choice]*

| ○＜1 |
| --- |
| ○1~3 |
| ○3~5 |
| ○＞5 |
