# Supplementary material for: The relationship between the psychological stress of adolescents in school and the prevalence of chronic low back pain: a cross-sectional study in China
Source: Child Adolesc Psychiatry Ment Health. 2019 Jun 17;13:24. doi: 10.1186/s13034-019-0283-2 (PMC6580587; doi:10.1186/s13034-019-0283-2)
Supplement: Supplementary file 2 — Additional file 2. Questionnaire (the Original version). [file 13034_2019_283_MOESM2_ESM.docx]

**大学生心理精神状况与颈肩腰背痛的相关性研究**

**第一部分 基本信息**

请问您的身高是（以cm为单位）_________ 体重是（以kg为单位）_________
您的年龄是_________ [填空题] *

请问您的性别是？ [单选题] *

| ○男 | ○女 |
| --- | --- |

请问您在进入大学前的常住地是? [单选题] *

| ○北上广深 |
| --- |
| ○省会城市 |
| ○二线城市 |
| ○三线城市 |
| ○农村 |

请问您的大学和年级是？ [填空题] *

_________________________________

请问您的专业是？ [单选题] *

| ○工 | ○文 | ○理 | ○农 |
| --- | --- | --- | --- |
| ○商 | ○医 | ○其他 |  |

请问您就学期间住宿情况是？ [单选题] *

| ○学校宿舍 |
| --- |
| ○校外租赁 |
| ○住自己或亲友家 |

**第二部分 慢性疼痛及其拓展**

（剧烈运动后出现下列不适症状的除外）
几乎不：<一月1次
偶尔：一月1-3次
有时：一周1-3次
经常：一周>3次
总是：每天

您感到颈肩痛的频率是 [单选题] *

| ○几乎不 |
| --- |
| ○偶尔 |
| ○有时 |
| ○经常 |
| ○总是 |

您感到腰背痛的频率是 [单选题] *

| ○几乎不 |
| --- |
| ○偶尔 |
| ○有时 |
| ○经常 |
| ○总是 |

请问您每次出现上述症状时的强度如何？ [单选题] *

| ○严重影响正常生活 |
| --- |
| ○轻微影响正常生活 |
| ○不影响正常生活 |
| ○没有上述状况 |

请问您的吸烟频率？ [单选题] *

| ○几乎不 |
| --- |
| ○偶尔 |
| ○有时 |
| ○经常 |
| ○总是 |

请问您的饮酒频率？ [单选题] *

| ○几乎不 |
| --- |
| ○偶尔 |
| ○有时 |
| ○经常 |
| ○总是 |

一天内，您一般在工作学习坐多长时间？（累计总时长） [单选题] *

| ○<1h |
| --- |
| ○1h-3h |
| ○3h-5h |
| ○5h-10h |
| ○>10h |

**第三部分  心理健康**
 （填写者可以通过此部分对自己的心理健康状况进行评估，评估结果将在问卷填写结束后显示。）

在您最近一段时间（近三个月）的生活中，您是否感到以下状况并根据状况与描述的符合程度选择对应的选项

无——没有这种状况 
轻度——有这种状况但是比较轻微 
中度——有这种状况且较明显
相当重——有这种状况并且影响正常生活
严重——有这种状况并且严重影响正常生活

对性取向对象的的兴趣减退 [单选题] *

| ○无 | ○轻度 | ○中度 | ○相当重 | ○严重 |
| --- | --- | --- | --- | --- |

感到自己的精力下降 [单选题] *

| ○无 | ○轻度 | ○中度 | ○相当重 | ○严重 |
| --- | --- | --- | --- | --- |

有结束自己生命的想法 [单选题] *

| ○无 | ○轻度 | ○中度 | ○相当重 | ○严重 |
| --- | --- | --- | --- | --- |

容易哭泣 [单选题] *

| ○无 | ○轻度 | ○中度 | ○相当重 | ○严重 |
| --- | --- | --- | --- | --- |

感到受骗，中了圈套或有人想抓住您 [单选题] *

| ○无 | ○轻度 | ○中度 | ○相当重 | ○严重 |
| --- | --- | --- | --- | --- |

经常责怪自己 [单选题] *

| ○无 | ○轻度 | ○中度 | ○相当重 | ○严重 |
| --- | --- | --- | --- | --- |

感到孤独 [单选题] *

| ○无 | ○轻度 | ○中度 | ○相当重 | ○严重 |
| --- | --- | --- | --- | --- |
|  |  |  |  |  |

感到苦闷 [单选题] *

| ○无 | ○轻度 | ○中度 | ○相当重 | ○严重 |
| --- | --- | --- | --- | --- |

过分担忧 [单选题] *

| ○无 | ○轻度 | ○中度 | ○相当重 | ○严重 |
| --- | --- | --- | --- | --- |

对事物不感兴趣 [单选题] *

| ○无 | ○轻度 | ○中度 | ○相当重 | ○严重 |
| --- | --- | --- | --- | --- |

感到前途没有希望 [单选题] *

| ○无 | ○轻度 | ○中度 | ○相当重 | ○严重 |
| --- | --- | --- | --- | --- |

感到任何事情都很困难 [单选题] *

| ○无 | ○轻度 | ○中度 | ○相当重 | ○严重 |
| --- | --- | --- | --- | --- |

感到自己没有什么价值 [单选题] *

| ○无 | ○轻度 | ○中度 | ○相当重 | ○严重 |
| --- | --- | --- | --- | --- |

神经过敏，心中不踏实 [单选题] *

| ○无 | ○轻度 | ○中度 | ○相当重 | ○严重 |
| --- | --- | --- | --- | --- |

无缘故发抖 [单选题] *

| ○无 | ○轻度 | ○中度 | ○相当重 | ○严重 |
| --- | --- | --- | --- | --- |

无缘无故地突然感到害怕 [单选题] *

| ○无 | ○轻度 | ○中度 | ○相当重 | ○严重 |
| --- | --- | --- | --- | --- |

心跳得很厉害 [单选题] *

| ○无 | ○轻度 | ○中度 | ○相当重 | ○严重 |
| --- | --- | --- | --- | --- |

一阵阵恐惧或惊恐 [单选题] *

| ○无 | ○轻度 | ○中度 | ○相当重 | ○严重 |
| --- | --- | --- | --- | --- |

感到坐立不安心神不定 [单选题] *

| ○无 | ○轻度 | ○中度 | ○相当重 | ○严重 |
| --- | --- | --- | --- | --- |

感到害怕 [单选题] *

| ○无 | ○轻度 | ○中度 | ○相当重 | ○严重 |
| --- | --- | --- | --- | --- |

感到熟悉的东西变成陌生或不像是真的 [单选题] *

| ○无 | ○轻度 | ○中度 | ○相当重 | ○严重 |
| --- | --- | --- | --- | --- |

感到要很快把事情做完 [单选题] *

| ○无 | ○轻度 | ○中度 | ○相当重 | ○严重 |
| --- | --- | --- | --- | --- |

头脑中有不必要的想法或字句盘旋。 [单选题] *

| ○无 | ○轻度 | ○中度 | ○相当重 | ○严重 |
| --- | --- | --- | --- | --- |

忘记性大。 [单选题] *

| ○无 | ○轻度 | ○中度 | ○相当重 | ○严重 |
| --- | --- | --- | --- | --- |

担心自己的衣饰整齐及仪态的端正。 [单选题] *

| ○无 | ○轻度 | ○中度 | ○相当重 | ○严重 |
| --- | --- | --- | --- | --- |

感到难以完成任务。 [单选题] *

| ○无 | ○轻度 | ○中度 | ○相当重 | ○严重 |
| --- | --- | --- | --- | --- |

做事必须做得很慢以保证做得正确。 [单选题] *

| ○无 | ○轻度 | ○中度 | ○相当重 | ○严重 |
| --- | --- | --- | --- | --- |

做事必须反复检查。 [单选题] *

| ○无 | ○轻度 | ○中度 | ○相当重 | ○严重 |
| --- | --- | --- | --- | --- |

难以作出决定。 [单选题] *

| ○无 | ○轻度 | ○中度 | ○相当重 | ○严重 |
| --- | --- | --- | --- | --- |

脑子变空了。 [单选题] *

| ○无 | ○轻度 | ○中度 | ○相当重 | ○严重 |
| --- | --- | --- | --- | --- |

不能集中注意。 [单选题] *

| ○无 | ○轻度 | ○中度 | ○相当重 | ○严重 |
| --- | --- | --- | --- | --- |

必须反复洗手、点数目或触摸某些东西。 [单选题] *

| ○无 | ○轻度 | ○中度 | ○相当重 | ○严重 |
| --- | --- | --- | --- | --- |

责怪别人制造麻烦。 [单选题] *

| ○无 | ○轻度 | ○中度 | ○相当重 | ○严重 |
| --- | --- | --- | --- | --- |

感到大多数人都不可信任。 [单选题] *

| ○无 | ○轻度 | ○中度 | ○相当重 | ○严重 |
| --- | --- | --- | --- | --- |

感到有人在监视您谈论您。 [单选题] *

| ○无 | ○轻度 | ○中度 | ○相当重 | ○严重 |
| --- | --- | --- | --- | --- |

有一些别人没有的想法或念头。 [单选题] *

| ○无 | ○轻度 | ○中度 | ○相当重 | ○严重 |
| --- | --- | --- | --- | --- |

别人对您的成绩没有作出恰当的评价。 [单选题] *

| ○无 | ○轻度 | ○中度 | ○相当重 | ○严重 |
| --- | --- | --- | --- | --- |

感到别人想占您的便宜。 [单选题] *

| ○无 | ○轻度 | ○中度 | ○相当重 | ○严重 |
| --- | --- | --- | --- | --- |

对旁人责备求全 [单选题] *

| ○无 | ○轻度 | ○中度 | ○相当重 | ○严重 |
| --- | --- | --- | --- | --- |

和异性相处时感到害羞不自在 [单选题] *

| ○无 | ○轻度 | ○中度 | ○相当重 | ○严重 |
| --- | --- | --- | --- | --- |

您的感情容易受到伤害 [单选题] *

| ○无 | ○轻度 | ○中度 | ○相当重 | ○严重 |
| --- | --- | --- | --- | --- |

感到别人不理解您、不同情您 [单选题] *

| ○无 | ○轻度 | ○中度 | ○相当重 | ○严重 |
| --- | --- | --- | --- | --- |

感到人们对您不友好，不喜欢您 [单选题] *

| ○无 | ○轻度 | ○中度 | ○相当重 | ○严重 |
| --- | --- | --- | --- | --- |

感到比不上他人 [单选题] *

| ○无 | ○轻度 | ○中度 | ○相当重 | ○严重 |
| --- | --- | --- | --- | --- |

当别人看着您或谈论您时感到不自在 [单选题] *

| ○无 | ○轻度 | ○中度 | ○相当重 | ○严重 |
| --- | --- | --- | --- | --- |

感到对别人神经过敏 [单选题] *

| ○无 | ○轻度 | ○中度 | ○相当重 | ○严重 |
| --- | --- | --- | --- | --- |

感到公共场合吃东西很不舒服 [单选题] *

| ○无 | ○轻度 | ○中度 | ○相当重 | ○严重 |
| --- | --- | --- | --- | --- |

**第四部分 其他压力**

请问您是否对未来有清晰的规划？ [单选题] *

| ○没有规划 | ○有一些模糊想法 | ○有大体方向 | ○有清晰规划 |
| --- | --- | --- | --- |

您对您当前就读学校的满意程度？ [单选题] *

| ○非常满意 | ○较满意 | ○一般 | ○不太满意 | ○不满意 |
| --- | --- | --- | --- | --- |

您对您当前就读专业的满意程度？ [单选题] *

| ○非常满意 | ○较满意 | ○一般 | ○不太满意 | ○不满意 |
| --- | --- | --- | --- | --- |

您与同学相处融洽吗？ [单选题] *

| ○非常融洽，几乎没有冲突 | ○一般，偶尔有冲突 | ○较差，经常发生冲突 |
| --- | --- | --- |

您是否与家人（父母、兄弟姐妹）相处融洽？ [单选题] *

| ○非常融洽，几乎没有冲突 | ○一般，偶尔有冲突 | ○较差，经常发生冲突 |
| --- | --- | --- |

您是否觉得父母对您的期望很高 [单选题] *

| ○是 | ○否 |
| --- | --- |

您认为您平常的生活费用水平如何？ [单选题] *

| ○富余 | ○平衡 | ○相对不足 | ○短缺 |
| --- | --- | --- | --- |

您是否难以适应现在的生活节奏？ [单选题] *

| ○是 | ○否 |
| --- | --- |

您的兴趣爱好有多少？（例如绘画、摄影、电影、读书等等） [单选题] *

| ○一到两个 | ○三到五个 | ○五个以上 |
| --- | --- | --- |

您是否觉得时间紧迫，不够用 [单选题] *

| ○是 | ○否 |
| --- | --- |

您难以入睡或睡眠状况不好的情况发生的频率是： [单选题] *

| ○几乎每天 | ○每周四到五次 | ○每周一两次 | ○几乎不 |
| --- | --- | --- | --- |

您参加了多少社团或学生组织？ [单选题] *

| ○一个 | ○两个 | ○三个以上 | ○无 |
| --- | --- | --- | --- |

您认为 您的情感生活状态如何？ [单选题] *

| ○非常好 | ○较好 | ○一般 | ○较差 | ○非常差 |
| --- | --- | --- | --- | --- |

您是否觉得身边大多数人比你优秀、努力？ [单选题] *

| ○是 | ○否 |
| --- | --- |

您是否害怕不能达到自己的目标？ [单选题] *

| ○是 | ○否 |
| --- | --- |

您是否有脱发的症状或迹象？ [单选题] *

| ○是 | ○否 |
| --- | --- |

您感觉学习上同学之间竞争情况如何？ [单选题] *

| ○非常激烈 | ○比较激烈 | ○一般 | ○没有感觉 | ○不关心 |
| --- | --- | --- | --- | --- |

课余时间您主要在什么地方活动？ [单选题] *

| ○寝室 | ○图书馆、自习室 | ○体育场 | ○校外 |
| --- | --- | --- | --- |

你认为自己的成绩是否理想？（平心而论，不用谦虚） [单选题] *

| ○非常理想 | ○较理想 | ○一般 | ○较不理想 | ○很不理想 |
| --- | --- | --- | --- | --- |

您是否在学习之余有足够时间参加娱乐活动？ [单选题] *

| ○是 | ○否 |
| --- | --- |

您每周运动的频率是： [单选题] *

| ○＜1次 |
| --- |
| ○1~3次 |
| ○3~5次 |
| ○＞5次 |
